# How to Use the Ophthalmoscope

**Published:** 1878-01

**Authors:** 


How to Use the Ophthalmoscope. Being elementary instructions in Opthalmoscopy, arranged for the use of students, with thirty fine engravings. By Edgar A. Browne, Surgeon to Liverpool Eye and Ear Infirmary, etc. Philadelphia; Henry C. Lea. 1877.

This is a duodecimo volume of 120 pages, with splendidly executed plates, and, taken altogether, makes a very fine appearance for so small a book.

